# Modernization of Golgi staining techniques for high-resolution, 3-dimensional imaging of individual neurons

**DOI:** 10.1038/s41598-018-37377-x

**Published:** 2019-01-15

**Authors:** Katlijn Vints, Dorien Vandael, Pieter Baatsen, Benjamin Pavie, Frank Vernaillen, Nikky Corthout, Vasily Rybakin, Sebastian Munck, Natalia V. Gounko

**Affiliations:** 1 VIB-KU Leuven Center for Brain & Disease Research, Electron Microscopy Platform & VIB-Bioimaging Core, O&N4 Herestraat 49 box 602, 3000 Leuven, Belgium; 2 KU Leuven Department of Neurosciences, Leuven Brain Institute, O&N4 Herestraat 49 box 602, 3000 Leuven, Belgium; 3 VIB-KU Leuven Center for Brain & Disease Research, Light Microscopy Expertise Unit & VIB Bioimaging Core, O&N4 Herestraat 49 box 602, 3000 Leuven, Belgium; 4 VIB Bioinformatics Core, Rijvisschestraat 126 3R, 9052 Gent, Belgium; 5 Rega Institute, Department of Microbiology and Immunology KU Leuven, Herestraat 49 box 1044, 3000 Leuven, Belgium

## Abstract

Analysis of neuronal arborization and connections is a powerful tool in fundamental and clinical neuroscience. Changes in neuronal morphology are central to brain development and plasticity and are associated with numerous diseases. Golgi staining is a classical technique based on a deposition of metal precipitate in a random set of neurons. Despite their versatility, Golgi methods have limitations that largely precluded their use in advanced microscopy. We combined Golgi staining with fluorescent labeling and tissue clearing techniques in an Alzheimer’s disease model. We further applied 3D electron microscopy to visualize entire Golgi-stained neurons, while preserving ultrastructural details of stained cells, optimized Golgi staining for use with block-face scanning electron microscopy, and developed an algorithm for semi-automated neuronal tracing of cells displaying complex staining patterns. Our method will find use in fundamental neuroscience and the study of neuronal morphology in disease.

## Introduction

Classical histological staining techniques used in neuroscience, such as Nissl stain and many others, indiscriminately visualize all cells or structures of interest. Cell type-specific stains, including antibodies, reveal highly convoluted and entangled networks of axons, dendrites and cell bodies, often making it impossible to fully outline individual neurons or to reliably trace neurites. More recently, several techniques have been developed to allow visualization of single neurons, mostly with the use of advanced fluorescent techniques or genetic labeling methods^1,2^. These methods tend to be costly and heavily rely on complex instrumentation and skills. One technique, hailing from the golden age of histology, stands out in that it reveals subsets of cells, rather than all cells of the same type, and works in an all-or-nothing fashion, without much dynamic range, thereby producing images of remarkable contrast and clarity. The “black reaction” method, developed by Camillo Golgi in the late XIX century and progressively refined ever since, is based on the impregnation of neural tissue with heavy metal precipitate^3,4^.

In contrast to tracing methods based on gene delivery and genetic manipulations^5,6^, Golgi staining does not require special skills or expensive equipment, nor is it costly. In its original form, the Golgi method involves sequential incubation of tissue fragments in solutions of potassium dichromate and silver nitrate, followed by sectioning for light microscopy (LM). Later refinements sought to use chemicals other than salts of silver, e.g. mercury salts, for increased contrast and accelerated staining^7–9^.

The Golgi method was instrumental for many groundbreaking advances in neurobiology, such as the discovery of dendritic spines^10^. Today, Golgi staining techniques are still widely used in research and clinical diagnostics^11^, but they are incompatible with further studies of the subcellular, organellar, architecture of labeled neurons with electron microscopy (EM) due to the formation of large, electron-dense silver deposits, which mask ultrastructural details. The method has been adapted for electron microscopy by replacing silver salts with those of gold, resulting in far smaller particles often deposited at the periphery of neurons^12,13^.

In this report, we combined two Golgi techniques, the original method and the Cox variation, with a substantially accelerated tissue clearing method, and adapted the original Golgi staining for use with block face scanning electron microscopy (BF-SEM)^14,15^. We describe the first successful use of a Golgi-based staining technique for tracing neurons over their entire length with preservation of ultrastructural details and a reliable algorithm for semi-automated neuronal tracing in Golgi-stained material. We further use a combination of Golgi staining, fluorescent labeling, and tissue clearing to visualize spatial relationships between entire neurons and amyloid plaques in an Alzheimer’s disease (AD) model.

## Results and Discussion

### Compatibility of Golgi-Cox and original Golgi staining with light microscopy

**Figure 1:**
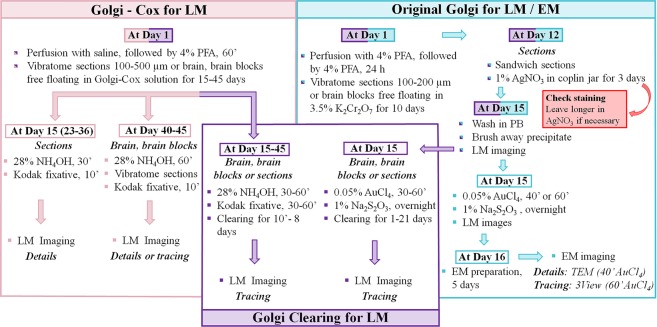
Adaptation of Golgi and Golgi-Cox staining for high-resolution LM and EM analysis. Pink, workflow for Golgi-Cox staining for light microscopy. Mice are perfused with PFA at day 1. After postfixation for 1 h in the same fixative, vibratome sections (100–500 µm) of the brain are made. Thin section (100 µm) could be impregnated with a sufficient number of neurons after 15 days in Golgi-Cox solution. Thick sections (150–500 µm) require 22–36 days, and whole brain or brain blocks, 40–45 days. The ammonium hydroxide step is made at day 15 for thin sections, day 23–36 for thick sections, or day 40–45 for the brain or brain blocks. Blue, workflow for original Golgi staining for light and electron microscopy. Mice are perfused as in above, and samples are postfixed for 24 h, followed by 10 days of impregnation with potassium dichromate. From day 12, samples are submitted to silver nitrate solution for at least 3 days, followed by gold toning for 40–60 minutes. Sections can be imaged by light microscopy from 15 days on. The EM preparation and embedding procedures take another 5–6 days. Purple, workflow for tissue clearing upon Golgi-Cox and original Golgi staining. For brain or large brain blocks after Golgi-Cox staining, the duration of clearing is 8 days, for hippocampi overnight, for thick (300–500 µm) sections between 2 h and overnight, and for thin sections 10 min. Original Golgi-stained sections and brain blocks required longer clearing procedures, 1 and 21 days respectively. See text for details. Abbreviations: EM, electron microscopy; LM, light microscopy; PFA, paraformaldehyde; PB, phosphate buffer; TEM, transmission electron microscopy.

**Figure 2:**
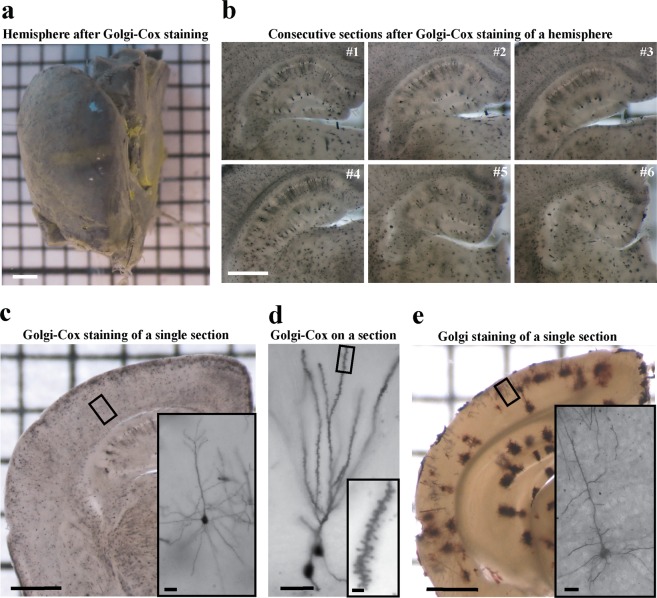
Golgi-Cox and Original Golgi for LM. (**a**) Half of a mouse brain after 44 days of Golgi-Cox staining, prior to sectioning. (**b**) Consecutive coronal vibratome sections (150 µm) obtained from the specimen shown in (**a**) display characteristic punctate staining patterns corresponding to bodies of stained neurons. Diffuse staining areas correspond to stained dendritic trees. (**c**) Visualization of neurites (insert) in a cell within a 300 µm coronal brain section after 22 days of Golgi-Cox staining. (**d**) Visualization of an impregnated hippocampal granule cell with dendritic spines (insert) in a 300 µm coronal brain section after 22 days of Golgi-Cox staining. (**e**) Visualization of neurites (insert) in a cell within a 200 µm section after original Golgi method. Scale bars: **a–c**, **e** = 1 mm, insert **c**, insert **e** = 20 µm, **d** = 40 µm, insert **d** = 2 µm.

We began by fine-tuning two Golgi staining techniques for light microscopy: Golgi-Cox and original Golgi. In the Golgi-Cox method, neurons are stained by free-floating the whole brain or brain sections in a solution of potassium chromate and potassium dichromate in the presence of mercury chloride (Fig. 1, left). In the original Golgi procedure, impregnation with silver chromate (Fig. 1, right) is performed with whole brains or larger tissue blocks, rather than sections. This is because the Golgi procedure causes the formation of electron-dense silver precipitate on the surface of the sample, making the microscopic analysis of sections very difficult. To identify optimal staining conditions, we used coronal vibratome sections of the mouse brain, 100–500 μm thick, as well as larger fragments, such as an entire hippocampus, halves of the entire mouse brain without cerebellum, and halves of the cerebellum. We observed reproducible impregnation of neurons in samples from paraformaldehyde-perfused animals following both Golgi-Cox and original Golgi techniques (Fig. 2). Golgi-Cox staining of brain halves (Fig. 2a) labeled randomly distributed individual neurons in consecutive 150 µm vibratome sections (Fig. 2b). With 300 µm coronal vibratome sections, the Golgi-Cox procedure did not result in the formation of surface precipitates (Fig. 2c,d), whereas some precipitation was noticeable after original Golgi staining (Fig. 2e and Supplementary Video 1). The optimal duration of impregnation for thin vibratome sections (100 µm) was 15 days, for thick sections (150–500 µm) 22–36 days, for brain blocks 40–45 days, and for cerebella 45 days in Golgi-Cox solution (Fig. 1). Shorter incubations (e.g. 8 or 14 days) yielded noticeable discontinuities along dendrites and less impregnated neurons (not shown). Both techniques successfully resolved dendritic trees by light microscopy (Fig. 2c,e inserts). For example, the Golgi-Cox method easily resolved individual dendritic spines in granule cells of the hippocampus (Fig. 2d, insert). It has been reported before^9,16,17^ that paraformaldehyde fixation may affect impregnation quality when the Golgi-Cox protocol is used. For example, the brain perfusion-fixed with 4% paraformaldehyde and post-fixed for 24 h yields high quality neuronal staining with Golgi-Cox impregnation for 14 days^16^. However, shorter impregnation with Golgi-Cox, e.g. 48 h at 37 °C, leads to a dramatic redistribution of the staining pattern from neuronal to glial cells^17^. In this protocol, we used 10-minute paraformaldehyde perfusion followed by a very short immersion post-fixation (1 h). The duration of impregnation in Golgi-Cox solution of at least one week increased selectivity of neuronal staining. After two weeks of impregnation, very fine details, such as spines of hippocampal granular cells, could be easily resolved (Fig. 2d).

An important limitation of the original Golgi technique is the formation of silver precipitates on the surface of the sample. Consequently, as mentioned above, blocks of tissue are usually stained and then sectioned to remove the surface precipitate. Impregnation, however, renders the tissue brittle and difficult to section^18^. We sought to minimize the effects of surface precipitation so as to be able to section the tissue prior to staining. After incubation of free-floating brain sections in potassium dichromate, sandwiching the sections between glass slides and coverslips during silver nitrate staining and rinsing^18^, we were able to carefully remove all surface precipitate using a small pony/goat hair painting brush (Fig. 1, and Supplementary Video 1).

Our results suggested that Golgi-Cox staining achieves higher resolution and lower background than the original Golgi method (compare Fig. 2c,e), and yields no noticeable precipitation (Fig. 2c,d).

### Imaging Golgi-stained neurons in cleared mouse brain tissue

We next explored the possibilities of using these two Golgi protocols for 3D light microscopic visualization of entire neurons morphology without sectioning. This necessitates the use of tissue blocks or thick sections. Due to light scattering, conventional imaging by multiphoton microscopy is limited to the depth of approximately 1 mm into tissue. New optical clearing methods use organic solvents to render tissues transparent, thereby reducing light scattering and allowing for considerably deeper imaging^1,19–21^. Multiple clearing techniques, such as CLARITY, iDISCO, and CUBIC^21^ are potentially suitable for combining with Golgi impregnation. At the light microscopy level, a combination of Golgi-Cox, but not original Golgi, with CUBIC and CLARITY clearing protocols has been recently published^22^. In that study, neurons were visualized in their entirety after a 48-hour clearing procedure.

**Figure 3:**
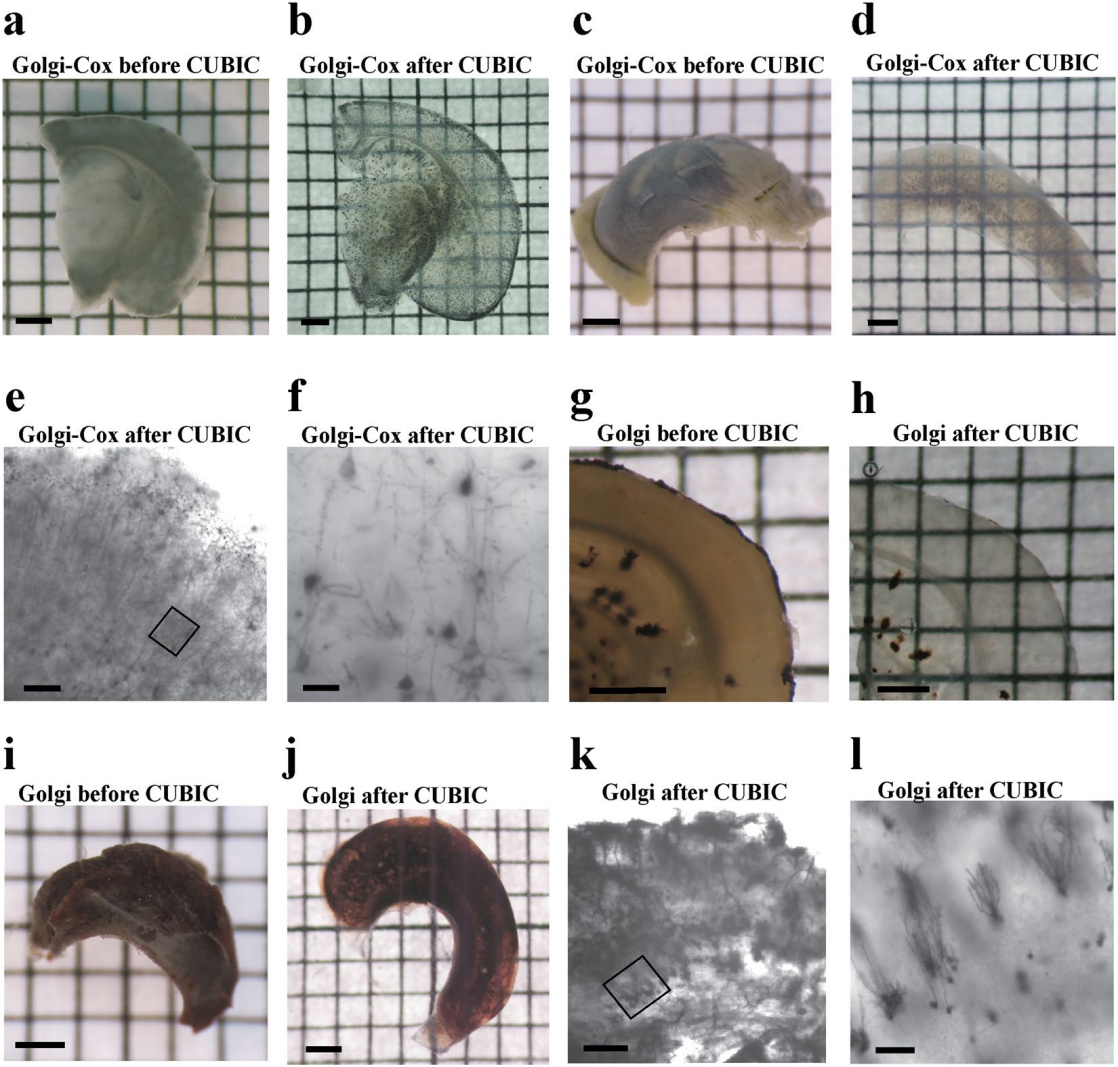
Tissue Clearing after Golgi-Cox and Original Golgi Staining. (**a**,**b**), 500 µm coronal brain section following Golgi-Cox impregnation (36 days) before clearing (**a**), and after clearing (**b**) overnight with modified CUBIC method. (**c**,**d**) Golgi-Cox-impregnated (29 days) whole hippocampus before (**c**) and after clearing (**d**) with modified CUBIC method overnight (**d**). (**e**,**f**) Cortical neurons after Golgi-Cox impregnation (43 days) and clearing for 24 h in CUBIC, imaged with a Nikon spinning-disk confocal microscope. (**f**) High magnification image of impregnated pyramidal neurons. (**g**,**h**) Original Golgi-impregnated 200 µm coronal brain section (**g**), and the same section after 24 h of CUBIC clearing (**h**). (**I,j**) Original Golgi impregnated whole hippocampus before CUBIC (**i**) and after 15 days of CUBIC protocol (**j**). (**k**,**l**), Original Golgi-stained, CUBIC-cleared hippocampus imaged with a Bioptonics OPT device, showing whole hippocampus tomography (**k**) and a high magnification of several granule cells (**l**). Scale bars: **a**–**d**, **g–j** = 1 mm; **e**, **k** = 200 µm, **f** = 40 µm, **l** = 100 µm.

We applied Golgi-Cox and original Golgi staining to 200 μm-, 500 μm-thick coronal sections of the brain, whole hippocampi, brain hemispheres without hippocampus, and halves of cerebellum, and then subjected stained samples to clearing protocols (Figs 1 and 3). We found that a modified CUBIC protocol^23^ showed optimal results with both Golgi-Cox (Fig. 3a–f) and original Golgi staining (Fig. 3g–l), readily visualizing dark neurons and providing good tissue transparency.

**Figure 4:**
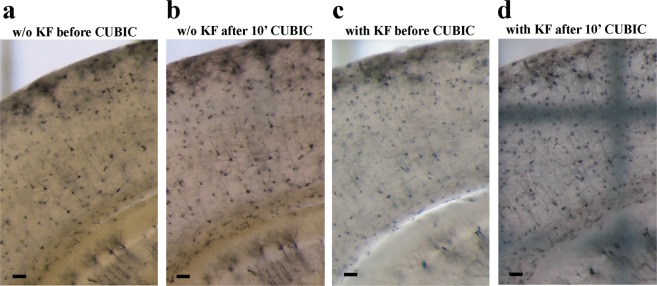
Optimization of Golgi-Cox procedure in combination with CUBIC. (**a**,**b**) Golgi-Cox-impregnated 150 µm coronal brain section before (**a**) and after (**b**) CUBIC protocol without Kodak fixative. (**c**,**d**) Similar samples treated with Kodak fixative before CUBIC protocol, before (**c**) and after clearing (**d**). Note a substantially accelerated tissue clearing compared to (**b**). Scale bars: 100 = µm.

Golgi-Cox stained 500 μm coronal sections (Fig. 3a) showed very good clearance after being subjected to the CUBIC protocol for as little as overnight (Fig. 3b), and 200 µm coronal sections after 10 minutes (not shown). Similarly, Golgi-Cox labeled hippocampus (Fig. 3c vs. 3d), and cortex (Fig. 3e,f) cleared very well using CUBIC clearing. The clearing of larger Golgi-Cox stained fragments required significantly longer incubations, e.g. overnight for hippocampi (Fig. 3d), 24 h for entire cortex (Fig. 3e,f), and 4 days for halves of the cerebellum (not shown). We obtained clearing throughout entire original Golgi labeled coronal sections and hippocampi with the CUBIC procedure (Fig. 3g–l). The clearing of original Golgi stained 150 μm sections (Fig. 3g vs. 3h) was accomplished in 24 hours, of whole hippocampi in 16 days (Fig. 3i vs. j and k,l), and of whole cerebella after 21 days (not shown). Clearing of Golgi-Cox stained samples could be dramatically accelerated by addition of an extra step to the CUBIC procedure, an incubation in Kodak photographic fixative (Fig. 1 and Fig. 4). Although the use of the fixative shortened the clearing procedure (compare Fig. 4a–d), it also accelerated the fading of the Golgi-Cox staining upon extended storage of specimens more than 2–3 days (not shown). It should be noted that the CUBIC procedure resulted in a certain degree of swelling of sections (compare Fig. 3a,b). The expansion of tissue due to the CUBIC treatment has been reported before^21,24^. Importantly, the swelling of samples does not result in alteration of the subcellular structure of neurons^24^. Nevertheless, tissue swelling during CUBIC is an important consideration which needs to be taken into account when planning experiments. It is highly recommended to process and image any control and experimental samples in parallel and within a reasonable timeframe, particularly in quantitative studies.

In conclusion, we were able to combine both Golgi-Cox and original Golgi staining procedure with tissue clearing and to significantly accelerate clearing. We achieved optimal results with a wide variety of samples, ranging from sections to large tissue fragments.

Next, we imaged neurons in 3D in Golgi-stained, cleared tissue without sectioning. We used optical projection tomography (OPT) imaging, which allows for the generation of 3D models from cleared biological samples by taking series of images of a rotating sample using conventional brightfield microscopy^25^. These images are projections through the sample that can be used to generate a section image using back projection. By imaging the whole hippocampus with OPT after original Golgi impregnation and clearing with CUBIC protocol, we were able to fully resolve the morphology of a dendritic tree of a dentate gyrus granule cell in 3D (Supplementary Video 2 and Fig. 3k,l). We obtained similar results with Golgi-Cox-stained cleared samples of brain hemispheres using a different method of 3D imaging, the Nikon spinning-disk confocal microscope with an objective suitable for deep imaging of tissues and whole organs upon clearing (Fig. 3e,f). We, therefore, succeeded in imaging entire neurons in 3D upon Golgi staining and tissue clearing.

### Combination of the Golgi-Cox method with fluorescent imaging

Accumulation of amyloid-β protein-containing plaques is a characteristic feature of Alzheimer’s disease (AD), but little is known about the direct consequences of plaque formation and growth for neuronal morphology. We combined Golgi-Cox staining with a specific fluorescent stain for amyloid plaques to simultaneously visualize plaques and neurites in the mouse cortex and hippocampus. We used brains of 25-month-old APP (APP^NL-G-F^) knock-in transgenic female mice^26^ and age-matched female wild type animals. At this age, APP mice accumulate amyloid-β plaques and recapitulate several AD-associated pathologies, including amyloid plaques, synaptic loss, and microgliosis and astrocytosis, especially in the vicinity of plaques^26^. Previous studies using this mouse model indicated that female mice are more prone to developing AD than males^27^. Earlier-onset pathology has been consistently seen in females across multiple APP transgenic models, but after 18 months of age, the accumulation of plaques plateaus at similar levels in both male and female mice^27,28^.

**Figure 5:**
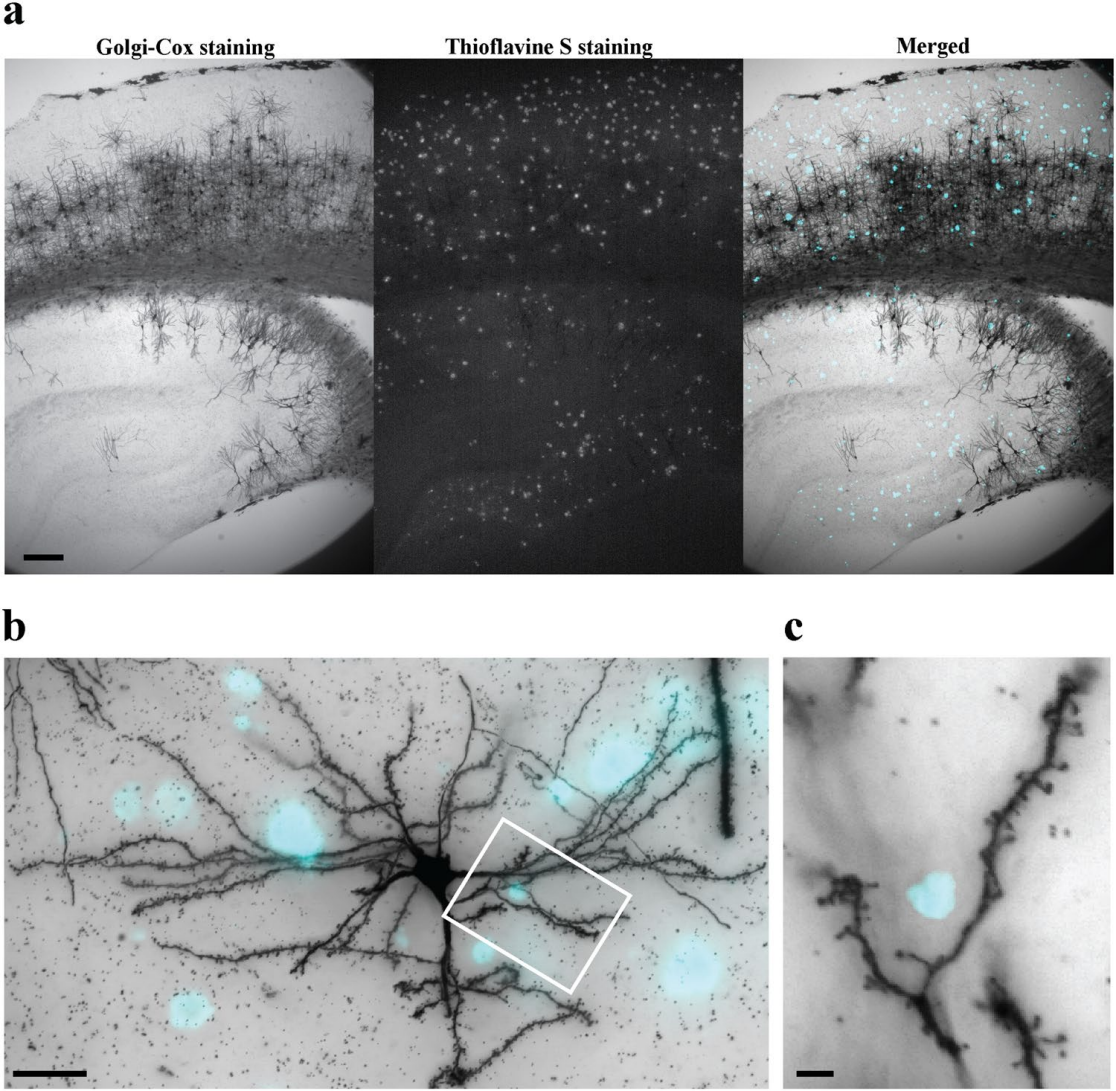
Golgi-Cox method and plaques staining in a mouse model of Alzheimer’s disease. (**a**) 200 µm coronal brain section of an APP knock-in mouse (APP^NL-G-F^) impregnated with the Golgi-Cox method and stained for amyloid-β plaques with thioflavin-S. Left, Golgi-Cox stain; middle, plaque stain; right, combined image, where plaques are shown in cyan. (**b**,**c**) High magnification of a z-projection image of an impregnated pyramidal cortical neuron and plaques (cyan). (**c**) Visualization of dendritic spines in the same impregnated cell as in (**b**), showing a plaque in very close proximity to a dendritic branch. Scale bars: **a** = 150 µm; **b** = 20 µm; **c** = 2 µm.

We submitted coronal brain sections and halves of brain of APP and wild-type mice to Golgi-Cox protocol (Fig. 5a, left), followed by thioflavin-S fluorescent staining^29^ of same sections to visualize plaques (Fig. 5a, middle). In APP mice, punctate thioflavin-S fluorescent staining was observed in the cortex and in the hippocampal region (Fig. 5a), and no staining was seen in the wild-type brain (see Supplementary Fig. S1). After CUBIC clearing, we imaged individual neurons in the plaque-containing cortex at high magnification (Fig. 5b) and observed efficient resolution of individual dendritic spines at light microscopy level (Fig. 5c). This experiment convincingly demonstrates that Golgi-Cox staining can be combined with fluorescent imaging and tissue clearing in a real-life scenario, for example, for a study of physical distortions of neuronal structure at different stages of AD by amyloid-β plaques.

### Original Golgi staining and correlative light and electron microscopy

Our ultimate goal was to perform correlative light and electron microscopy and to combine whole-cell morphology analyses with ultrastructural imaging of the same neurons. We chose to further develop the original Golgi method for EM. Mercury chloride, an essential component of the Golgi-Cox procedure, is not well suited for EM due to its tendency to sublimate in high vacuum, posing danger to both EM operators and instrumentation. Although Golgi-Cox staining in combination with EM has been reported, this was done by using a glutaraldehyde post-fixation step to immobilize mercury within the specimen and using ultrahigh-voltage electron microscopes operating at 2 MeV to selectively sublimate non-specifically deposited mercury^30^. These are both impractical, and, in addition, the resolution of the images obtained by Golgi-Cox combined with EM was not suitable for analyses of cellular ultrastructure. Therefore, for electron microscopy, we continued with the original Golgi technique.

**Figure 6:**
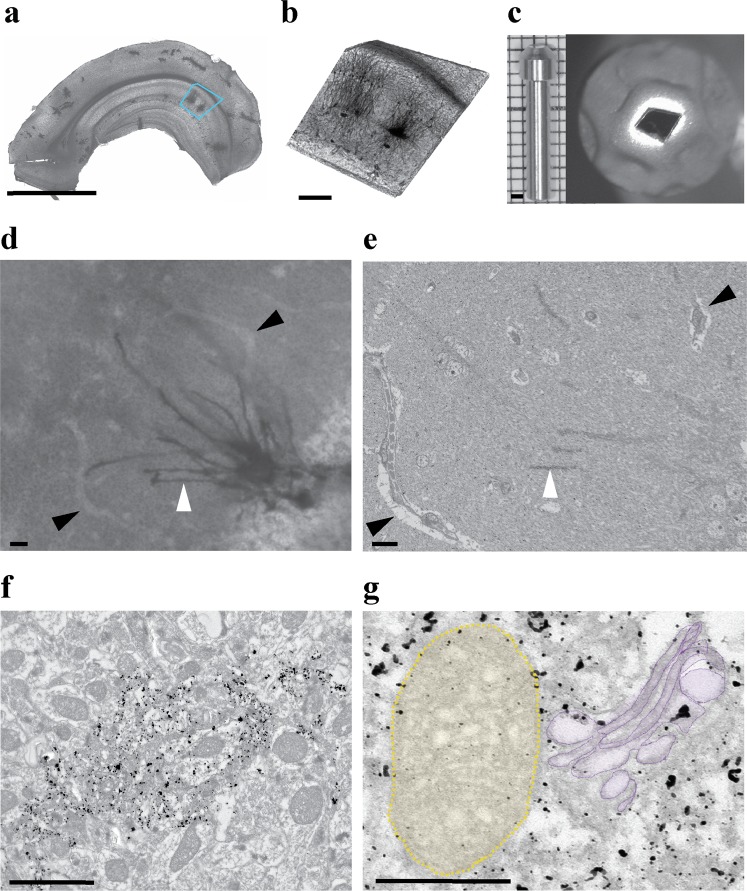
Original Golgi for EM details. (**a**) A 100 µm brain section impregnated with the original Golgi method and followed by a short gold toning procedure for 40 min. The blue box indicates the ROI. (**b**) ROI excised from the specimen shown in (**a**), prior to serial BF- SEM. (**c**) Block profile (left) with the cropped section as in (**b**) after EM protocol, glued to a pin (right) for serial BF-SEM. (**d**,**e**) The sample after 40 min gold toning, imaged with LM (**d**) and serial BF- SEM (**e**). Blood vessels are visible as lighter areas indicated by black arrowheads. A distinctive region of a cell of interest is indicated by a white arrowhead. (**f**,**g**) High magnification TEM micrographs of an impregnated neuron. The size of gold particles allows resolving the intracellular organelles of the impregnated neuron. (**g**) Details of neuronal ultrastructure after modified Golgi staining. Mitochondria are highlighted in yellow and Golgi apparatus in purple. Note the preservation and resolution of membranous and vesicular structures. Scale bars: **a** = 2 mm; **b** = 200 µm, **c** (left) = 1 mm; **d**, **e** = 20 µm; **f** = 2 µm; **g** = 200 nm.

**Figure 7:**
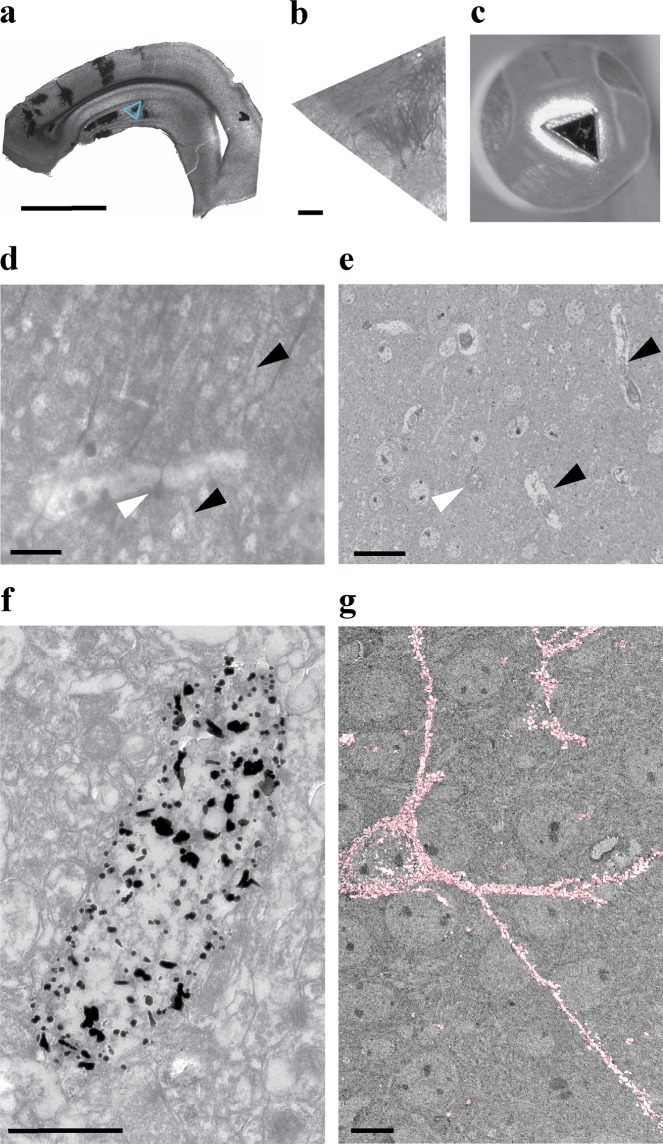
Original Golgi for EM tracing. (**a**) A 100 µm brain section impregnated with the original Golgi method and followed by an extended gold toning for 60 min. The blue box indicates the ROI. (**b**) ROI excised from the specimen shown in (**a**), prior to imaging. (**c**) Block profile with the cropped section as in (**a**) after EM protocol, glued to a pin for serial BF-SEM. (**d**,**e**) The sample after 60 min of gold toning, imaged with LM (**d**), and serial BF-SEM (**e**). Blood vessels are visible as lighter areas indicated by black arrowheads. A distinctive region of a cell of interest is indicated by a white arrowhead. (**f**,**g**) High magnification TEM micrograph of an impregnated neuron. Note large sized, electron-dense deposits clearly masking some of the ultrastructure but providing much higher contrast as compared to Fig. 6f. (**g**) The size of gold particles allows to easily follow an impregnated neuron via a 3D volume and to fully reconstruct it, as shown in pink. Scale bars: **a** = 2 mm; **b** = 200 µm; **d–e** = 20 µm; **f** = 2 µm; **g** = 10 µm.

We aimed to achieve a comparatively weak Golgi staining so as to minimize the interference with cellular ultrastructure, while still allowing for reliable identification and tracing of the stained cells by light and electron microscopy. The following workflow allowed to efficiently integrate LM and EM with Golgi (Figs 6, 7):

(i) Golgi sections were prepared for LM (Figs 6a, 7a); Neuron of interest were identified and imaged by LM (Figs 6b, 7b);
(ii) The gold toning procedure was performed;
(iii) LM was performed again to confirm the position of the neuron of interest, taking into account the patterns of blood vessels using DIC (Figs 6d, 7d) for future correlation with EM (Figs 6d,e, 7d,e). The high contrast of the Golgi staining and unique blood capillary patterns facilitate easy recognition of the same areas of interest with LM and EM imaging.
(iv) Osmication, en bloc staining with uranyl acetate and lead aspartate, dehydrate, and embedding in epoxy resin were performed, and specimens were glued to pins for BF-SEM (Figs 6c, 7c).

We modified the Fairén procedure^13,31^, which supplements Golgi staining with a gold toning procedure. Gold toning replaces silver chromate with gold chloride, reduces gold to its atomic state with oxalic acid, and finally removes silver nitrate with thiosulfate. By controlling the duration of the gold toning step, we could achieve the formation of either a very fine gold precipitate that did not obstruct ultrastructural details or larger size gold particles suitable for easy tracing. As shown in Fig. 6f, a short toning procedure resulted in the formation of small-sized gold particles that were easily recognizable and did not substantially mask subcellular details of impregnated neurons, such as mitochondria and the Golgi apparatus (Fig. 6g).

Extension of the gold toning step yielded larger gold deposits, facilitating easier identification and tracing of impregnated neurons in EM (Supplementary Videos 3 and 4), but also obscuring more of the ultrastructure, as illustrated in Fig. 7f. The longer gold step not only allowed to easily trace and reconstruct the impregnated neuron, but also to reconstruct its presynaptic connections, since the preservation of ultrastructure was still adequate (Fig. 7f,g and Supplementary Video 4). After imaging an impregnated hippocampal granule cell with BF-SEM and reaching the region of interest (e.g. a tertiary dendrite of an impregnated cell), we switched to conventional manual thin sectioning and imaging by conventional transmission electron microscopy (TEM) of the entire volume of the region of interest to achieve better resolution. The manual serial sectioning was performed due to a number of advantages over other imaging methods such as electron tomography. The potentially higher resolution of electron tomography can only be achieved in a restricted volume close to the tilt axis. Manual serial sectioning, on the other hand, allows wide areas of the sample to be observed at the same resolution at any given time, providing an important advantage in the investigation of neuronal wiring, where paths of neurites may be highly complex. Further, any section can be easily referenced again any number of times if necessary. The sections can be imaged by conventional TEM (80–120 kV), and a higher acceleration voltage microscope (200 kV) is not needed for imaging thin sections, as would be typical for electron tomography (400–600 nm). Finally, for the analysis of large volumes (more than 2 μm depth), such as those investigated and imaged here, an electron tomography imaging approach would still require serial thick sectioning, e.g. 5–6 sections to cover 2 μm of an area of interest. Taking into account the sectioning, imaging, and 3D assembly of individual tomograms, this can be logistically challenging.

Thereafter, we returned to serial BF-SEM to image the rest of the cell and combined all images from BF-SEM and TEM into a single stack (Supplementary Video 4). This approach allowed us to obtain and reconstruct *unlabeled* presynaptic connections to the impregnated neuron in any region of interest with high resolution and to combine these data with our semi-automatic tracing and reconstruction of the impregnated neuron. Therefore, Golgi-staining lends itself very well for correlative LM and EM, and silver and/or gold deposits are readily visible by both light and electron microscopy.

### 3D imaging and an algorithm for automatic detection and tracing of Golgi-stained neurons

Finally, we adapted the staining procedure for use with semi-automated image processing and developed an algorithm for tracing and reconstitution of Golgi-stained cells across multiple sections imaged with a BF-SEM. As Golgi staining for EM results in variable grain size, density, and distribution, the task of identifying and delineating positively stained neurons is not straightforward. Simple thresholding would result in unconnected groups of dots representing the gold grains. We applied mathematical morphology algorithms to connect the grains within the same neurons. Initial segmentation experiments suggested that a combination of simple preprocessing filtering followed by thresholding and morphological filter operations allowed to identify the outline of the stained area. The result of such segmentation can be used as input for an active contour-based algorithm to improve the detection of the shape of the neuron further. The automatic segmentation algorithm for tracing impregnated neurons in a 3D volume has been packaged as a plugin for ImageJ. We used the plugin to semi-automatically segment an individual stained neuron, as shown in Supplementary Video 3. Next, we applied this approach to image a cell throughout a 3D volume, followed by semi-automatic segmentation for tracing in 3D and reconstruction not only of an impregnated neuron but also of presynaptic boutons (Supplementary Video 4). Our method, therefore, allows not only to study individual cells but also to follow synaptic connections of impregnated cells.

Golgi methods are inexpensive, relatively easy to carry out, and can be easily adapted and used with various microscopic modalities. Here, we describe a series of new approaches designed to facilitate the application of Golgi-based methods for the study of neuronal morphology and connectivity using the most modern ultrastructural techniques. Our approaches visualize easily identifiable and traceable cells and preserve their ultrastructure for additional analyses. The combination of highly modified Golgi techniques with tissue clearing and fluorescent techniques allows for the visualization of well-spaced, easily identifiable cells in their entirety with unprecedented resolution and level of detail within their tissue context.

## Materials and Methods

### Animals

All animal experiments were approved by the KU Leuven Ethical Committee (protocol P138/2017) and were performed in accordance with the Animal Welfare Committee guidelines of the KU Leuven, Belgium. In total, fifty-seven 4–6-week old male and female C57BL/6 mice for Golgi-Cox and original Golgi, and two female APP^NL-G-F^ knock-in, 25 month-old transgenic mice with two age-matched wild type female controls were used. Animals were euthanized with a mixture of ketamine and xylazine as per institutional guidelines.

Mice were transcardially perfused with 10 ml of 0.9% sodium chloride (#S7653, Sigma-Aldrich, Belgium) in double distilled water (dH_2_O) for 10 min for Golgi-Cox, followed by 10 ml for 10 min of 4% PFA (#15714, EMS, USA) in 0.1 M phosphate buffer (PB), pH 7.4. For the original Golgi staining, the sodium chloride step was omitted. Brains were postfixed overnight for original Golgi or for 1 h for Golgi-Cox at 4 °C. In some experiments, 100–500 µm-thick coronal vibratome sections were made. Typically, for light microscopy imaging, the thickness of vibratome sections was between 100–500 µm, for electron microscopy between 100–200 µm, and for Golgi in combination with tissue clearing 150–500 µm.

### Golgi-Cox staining for LM

After three washes in 0.1 M PB, sections or tissue blocks were free floated in Golgi-Cox solution (see below) at room temperature (RT) for 15–45 days (see text for details), in the dark. Next, sections or blocks were washed two times for 5 min with dH_2_O, transferred into 28% ammonium hydroxide (#221228, Sigma-Aldrich, Belgium) in dH_2_O, and incubated for 30 min at RT with rotation. After washing two times for 5 min with dH_2_O, the sections were incubated in 15% Kodak Carestream Fixer solution (#P6557, Sigma-Aldrich, Belgium) in dH_2_O for 10 min at RT, followed by two 5 min washes with dH_2_O. Brain blocks after incubations in 28% ammonium hydroxide for 60 min and washing with dH_2_O were embedded into 3% agarose in dH_2_O, and 150–300 µm thick vibratome sections were made. The sections were incubated in 15% Kodak Carestream Fixer solution in dH_2_O for 10 min at RT, followed by mounting onto gelatin-coated microscopic slides, dried at RT, and covered with glass coverslips in Mowiol mounting medium (#81381-50 G, Sigma-Aldrich). Whole sections were imaged with a Zeiss Stereomicroscope, and cells/spines were imaged with a Zeiss Axioplan 2 widefield microscope using a 20x and 40x objective.

Golgi-Cox solution is a mixture of three components: (A) 5% potassium dichromate in dH_2_O (#P5271, Sigma-Aldrich, Belgium), (B) 5% mercury chloride (#KK04.1, Carl Roth GmbH, Germany) in dH_2_O dissolved with heating to 60 °C, and (C) 5% potassium chromate in dH_2_O (#HN33.1, Carl Roth GmbH, Germany). Firstly, 50 ml of RT solution A is mixed with 50 ml of RT solution B. Then, 40 ml of RT solution C is mixed with 100 ml of dH_2_O and slowly added to the mixture while stirring. After stirring for an additional 2–5 min, the solution is covered with aluminum foil and left at RT overnight, whereupon a red/yellow precipitate forms. Solution is carefully aspirated, leaving the precipitate and the surface layer undisturbed, passed through a 0.2 µm filter, and used within 20–30 days of preparation.

### Original Golgi staining for LM and EM

100–200 µm vibratome sections or brain blocks were washed three times with 0.1 M PB and incubated for ten days in 3.5% potassium dichromate in dH_2_O at RT in the dark, free floating. Then, 5–6 sections were collected with a soft painting brush (series 150, size 1; Talens, The Netherlands) onto microscopic glass slides and covered with 22 × 22 mm coverslips, corners of which had been dipped into commercial cyanoacrylate glue. The “sandwich” and the brain fragments were put into 250 ml Coplin jars filled completely with 1% silver nitrate (#RT210560, EMS, USA) in dH_2_O. After three days at 37 °C in the dark, the staining was checked with the use of a widefield light microscope and, if staining appeared insufficient, the incubation in 1% silver nitrate was extended.

The next steps apply to sections. After unmounting the sections from slides and washing them in 0.1 M PB, the precipitates were removed by gently scraping the surface of the sections with a painting brush in 0.1 M PB (Supplementary Video 1). The sections can be stored in 0.1 M PB at 4 °C until imaging with a widefield light microscope to select stained neurons. The whole sections were imaged with a Zeiss Elyra S.1 microscope using a 4x objective (Figs 6a and 7a). Sections were then cropped to the size of approx. 1 mm² around the neuron of interest, preferably in a way that allows for easy orientation of the cropped sample (Fig. 6a–c and Fig. 7a–c) and the areas of interest were imaged with Zeiss Elyra S.1 microscope using a 4x objective.

Cropped sections were washed three times in dH_2_O for 10 min, followed by incubation in 0.05% gold chloride (#RT16586, EMS, USA) in dH_2_O, at 4 °C in the dark with agitation for either 40 min (for better preservation of ultrastructural details) or 60 min (for easier tracing), and between 30–60 min for clearing step. Samples were washed again three times for 10 min with ice-cold dH_2_O, incubated for 10 min in ice-cold 0.5% oxalic acid (#241172, Sigma-Aldrich, Belgium), washed three times in ice-cold dH_2_O for 10 min, and incubated in 1% sodium thiosulfate (#RT21360, EMS, USA) at 4 °C with rotation overnight. Sections (z-stack) were imaged with a Zeiss Elyra S.1 microscope with differential interference contrast (DIC), using 4x and 40x objectives.

Samples were then stained with 1% osmium tetroxide (#19152, EMS, USA) and 1.5% potassium ferrocyanide (#455989, Sigma-Aldrich, Belgium) for 1 h, followed by 0.2% tannic acid (#21700, EMS, USA), and again 1% osmium tetroxide at RT for 30 min, with washes in dH_2_O between steps. Next, samples were incubated in 0.5% uranyl acetate (#22400, EMS, USA) in 25% methanol overnight at 4 °C and stained *en bloc* with Walton’s lead aspartate^32^ for 30 min at 60 °C. After washing with dH_2_O, samples were dehydrated in ascending ethanol series (30%, 50%, 70%, 80%, 95%, 100%), 10 min per step, at 4 °C. Samples were treated twice for 10 min each with propylene oxide at RT and infiltrated with hard Epon 812/propylene oxide mixtures. The next day, sections were flat embedded in hard composition of Epon 812 (#14120, EMS, USA) between two microscopic slides and ACLAR film (#50425, EMS, USA) and polymerized for 2 days at 60 °C.

For tracing impregnated neurons within the volume, samples gold-toned for 60 min were imaged with a Zeiss Sigma Variable Pressure scanning electron microscope (Zeiss, Germany) equipped with an en-suite ultramicrotome (Gatan, 3View, France). Flat-embedded sections were mounted on aluminum pin stubs (Gatan, France) with conductive epoxy (Circuit Works, #16043, Ted Pella, Germany). Neurons of interest could be located based on staining and LM imaging and the geometry of the adjacent blood vessels. After the impregnated neuron of interest was approached, serial sectioning was performed at 200 nm steps. This increment was chosen to facilitate the imaging of the whole neuronal volume required for tracing, at a reasonable speed. Additionally, sectioning larger specimens at z-steps below a certain threshold results in imaging artefacts arising from the accumulation of local negative charge when the penetration depth of the electron probe exceeds section thickness. Related to that and possibly due to resistive heating of the resin or other alterations of the resin, sectioning artefacts can occur, such as incomplete sectioning, or skipping of sections. We have not observed any obvious differences in dendritic morphology of impregnated neurons using 200 nm vs 100 nm (standard thickness for TEM) in pilot studies (data not shown). Smaller z-steps can be used if necessary but may require further optimization.

Serial electron microscopic images of half volume of an impregnated neuron were acquired at 1.3 kV with 200 × 200 µm field of view at 2 µs dwell time, 0.012 µm/pixel (the imaging time was 6 minutes per section). Then, to reconstruct synapses connected to the impregnated neuron (Supplementary Video 4), the block was removed from the microscope once the area of interest was identified for manual sectioning of 2–5 µm of impregnated neuron and after manual cutting returned to BF-SEM for imaged rest of the volume with same parameters. Images of BF-SEM were aligned using the Digital Micrograph Software (Gatan). We developed a semi-automatic algorithm for segmentation of imaged neurons across the stack (see below, and Supplementary Video 3). Reconstruction of segmented neurons was done using the Amira software (FEI/Thermo Fisher Scientific, France), segmentation and reconstruction of synapses to impregnated neuron was done by Amira software as well.

For observation of ultrastructural details of impregnated neurons with high resolution, blocks containing samples gold-toned for 40 min were removed from the microscope once the area of interest was identified using BF-SEM (Fig. 6). Serial ultrathin sections were generated manually using a Reichert Ultracut E ultramicrotome. All sections were collected as ribbons of 4–5 sections on triple slot grids (Ted Pella, Germany). Images were taken on a TEM microscope (JEM1400-LaB6, JEOL, Japan) operated at 80 kV. TEM was equipped with an Olympus SIS Quemesa 11 MP camera. Photographs were taken at various magnifications (0.6–20kx).

### Golgi clearing for LM using CUBIC method

After Golgi-Cox and Golgi staining, samples (sections or brain bocks) were clarified using CUBIC protocol. For Golgi-Cox-stained samples, tissue clearing was started after the incubation with 15% Kodak fixative, and for original Golgi-stained samples, after the 0.05% gold chloride for 30–60 min. Samples were washed three times in dH_2_O for 10 min, followed by incubation in the CUBIC solution: 250 g/l urea (#821527, ICN Biomedicals, USA), 250 g/l Quadrol (#122262, Sigma-Aldrich, Belgium), and 150 g/l Triton X-100 (#RT22140, EMS, USA), sequentially dissolved in dH_2_O with heating and stirring, and cooled to RT). Golgi-Cox-stained samples were incubated for 10 min - 8 days (see text) in a mixture of equal parts of CUBIC solution and dH_2_O, at 37 °C in the dark with agitation. Original Golgi-stained samples were submitted to the same CUBIC protocol and incubations continued until the desired level of tissue clearing was achieved, 1–21 days (see text).

Samples were imaged directly in clearing solutions. 3D light microscopy images were taken with a Bioptonics OPT device (Fig. 3k,l, and Supplementary Video 2) or a Nikon spinning-disk confocal with a CFI Plan Apo 10XC Glyc Objective for Clearing Applications (Fig. 3e,f). Whole samples were imaged with a Zeiss Stereomicroscope (Fig. 3a–d,g–j).

### Golgi-Cox and fluorescent staining for LM

Sections were impregnated with Golgi-Cox protocol as above. After the ammonium hydroxide step and washing two times for 5 min with dH_2_O, the sections were stained with 0.05% thioflavin-S (#T1892-25G, Sigma-Aldrich, Belgium) in 50% methanol for 20 min with gentle agitating in the dark. Samples were washed two times with dH_2_O for 5 min, incubated in Kodak fixative for 10 min, and washed two times before clearing with CUBIC protocol for 10 min. Sections were mounted in the last change of CUBIC solution and imaged with a Zeiss Axioplan 2 widefield fluorescence with 5x and 40x objective (Fig. 5a–c).

### An ImageJ plugin for segmentation of Golgi stained EM images

The GolgiStain tool is a plugin for ImageJ2 (http://imagej.net/) or Fiji2 (https://fiji.sc/), which can be downloaded at http://bioimagingcore.be/plugin/golgi-stain-1.2.0.jar. It works on all systems supported by ImageJ2. To install the latest version of GolgiStain, add the GolgiStain site to your list of ImageJ update sites as follows: (1) from the ImageJ menu select *Help ▶ Update*…, (2) then click on *Manage update sites*, (3) click *Add update site*, fill in http://sites.imagej.net/GolgiStain/ as URL, provide a name (e.g. GolgiStain), make sure the checkbox is checked, press *Close*, and finally (4) click *Apply changes* and restart ImageJ. An alternative installation method is to drag-and-drop the golgi-stain-1.2.0-.jar file into the ImageJ plugin directory; in this case, plugin updates will need to be performed manually as well.

#### Quick-start instructions

(1) Load the dataset into ImageJ. The dataset must be an 8 or 16 -bit grayscale image or an image stack. (2) Start the GolgiStain plugin from the menu: Plugins ▶ GolgiStain. The plugin window with input fields for the segmentation parameters will appear. (3) Adjust the segmentation parameters (see Supplementary Video 3). If the Preview checkbox is checked, a red overlay will appear showing the segmentation result. For image stacks, the slice to use for the segmentation preview can be specified with the *Slice #* for *Preview* input field. (4) Press OK to perform the segmentation on the image or the full image stack. If the *Mask Segmentation Only* checkbox is selected, each slice in the output will be a binary mask; otherwise, a multichannel output is generated with channel 1 being the original data and channel 2 being the mask.

The plugin performs image segmentation via a pipeline of simple operations. As the Golgi stain particles are very dense, they can be identified easily in the image via simple thresholding. Neighboring particles are then merged into contiguous regions via a combination of morphological closing and Gaussian blurring (morphological closing efficiently connects neighboring particles and closes holes, but results in hard jaggy region contours, a subsequent Gaussian blur followed by thresholding smoothens the region contours.). The resulting image is then thresholded once again to extract the binary masks for the stained regions. Finally, small isolated staining particles can be discarded by filtering the regions by area.

#### Description of segmentation parameters

- *Max Staining Particles Intensity*: The threshold value that will be used to decide which pixels are considered to be Golgi stain granules. Only pixels with intensity between 0 and the entered value are taken to be staining particles; all other pixels are “discarded”.
- *Close Radius (in pixels)*: The extent of the morphological closing. All particles that are within this distance from one another will be merged.
- *Blur Distance (pixels)*: The size of the Gaussian blur. Its effect is to smoothen the outline of the segmentation regions.
- *Min Segment Intensity*: The pixel intensity threshold used to extract the actual segmentation regions after the Gaussian blur. Small values will result in a segmentation where the region boundaries are too far from the staining particles, whereas too large values result in disconnected regions. Set this value to 1 initially while optimizing the other parameters. Once a reasonable segmentation is obtained, this value can then be increased little by little to bring the region boundaries sufficiently close to the staining particles.
- *Min Segment Size (in pixels)*: This is the minimum area (in a number of pixels) for a region to be allowed in the final segmentation output. It is used to discard tiny isolated, stained regions. It is best set to 0 initially to avoid accidentally suppressing relevant segmentation regions. Once all other parameters are optimized, this value can (optionally) be increased to remove, for example, regions that are suspected to be non-specific staining.
- *Segmentation Mask Only*: If this checkbox is checked, the output will be a binary segmentation mask. If it is not checked, the output will be a two-channel image (stack) where channel 1 is the original data and channel 2 the segmentation mask. The two-channel output is very convenient for inspecting the segmentation result, but it requires more computer memory. The binary output may also be preferable for further processing, such as 3D reconstructions.
- *Preview Opacity (in %)*: the percentage of opacity set to the preview overlay, the higher, the more opaque.
- *Slice # for Preview*: The number of the slice for which to preview the segmentation result. It is used for image stacks (>1 image).
- *Preview*: If this checkbox is checked the preview overlay will be recalculated and redrawn each time the user modifies a segmentation parameter and presses *Enter*.

#### Algorithm details

**Figure 8:**
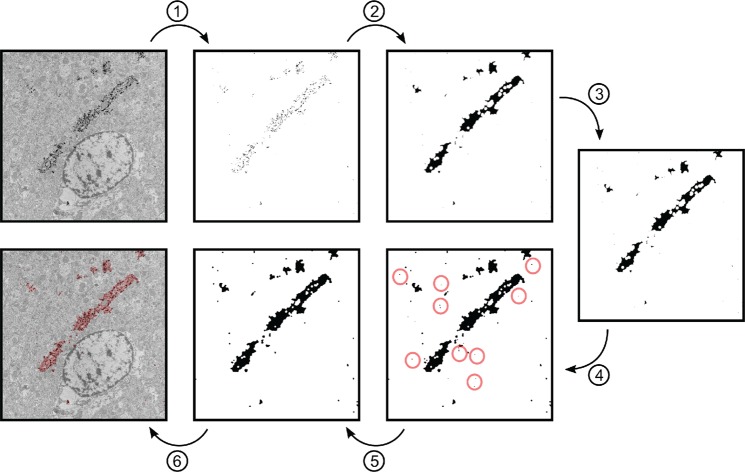
The segmentation pipeline for ImageJ. Dense Golgi-stained particles are detected in the image using a simple thresholding operation (1). The resulting discrete objects are then joined into contiguous regions using a morphological closing operation (2). To smoothen these regions, a Gaussian blur filter (3) is applied, followed by a second thresholding operation (4). Non-specific staining is removed by sorting the segmented regions by area and discarding regions with area under the user-specified threshold (5). The remaining regions represent the result of segmentation and are added as a transparent channel to the original image stack (6). Alternatively, they can be returned as a stack of binary segmentation masks.

The segmentation pipeline consists of the following sequence of image processing steps (Fig. 8).

1. An initial threshold is performed. All pixels between 0 and the threshold *T*_1_ value entered by the user are set to the maximum pixel intensity 2^b^ − 1, the others to 0. (b is the bit depth of the image: 8 for 8-bit images, 16 for 16-bit images)
  $$g(x,y)=\{\begin{array}{c}0\,{\rm{if}}\,f(x,y) > {T}_{1},\\ {2}^{b}-1\,{\rm{otherwise}}\end{array}$$
2. A morphological closing operation is applied on the binary image obtained in step 1, to connect the disjointed stained particles:
  $$A\cdot B=(A\oplus B)-B$$
  A is the binary input image, B is the circular structuring element with user-specified radius, and $A\cdot B$ is the filtered result.
3. A Gaussian blur is applied with the user-specified kernel size σ.
  $$G(x,y)=\frac{1}{2\pi {\sigma }^{2}}{e}^{-\frac{{x}^{2}+{y}^{2}}{2{\sigma }^{2}}}$$
4. A second threshold is then used to select from the blurred image those pixels that are considered to be positive staining. All pixels above the threshold value T_2_ are set to the maximum pixel intensity 2^b^ − 1. Otherwise, they are set to 0. (b is the bit depth of the image)
  $$g(x,y)=\{\begin{array}{c}0\,{\rm{if}}\,f(x,y) < {T}_{2},\\ {2}^{b}-1\,{\rm{otherwise}}\end{array}$$
5. The thresholded image is treated as a binary image of segmentation regions. The regions are filtered by size using the standard Particle Analysis tool from ImageJ. Regions with an area smaller than the user-specified minimum (circled in red in Fig. 8) are discarded.
6. Finally, the resulting segmentation regions are displayed either as a binary segmentation mask or as a transparent overlay on top of the original image.

## Supplementary information

Supplementary Info

Supplementary Video 1

Supplementary Video 2

Supplementary Video 3

Supplementary Video 4

golgi-stain-1.2.0 plugin for ImageJ

## Data Availability

The datasets generated during and/or analysed during the current study are available from the corresponding author on reasonable request.
