# Supplementary material for: Modernization of Golgi staining techniques for high-resolution, 3-dimensional imaging of individual neurons
Source: Sci Rep. 2019 Jan 15;9:130. doi: 10.1038/s41598-018-37377-x (PMC6333844; doi:10.1038/s41598-018-37377-x)
Supplement: Supplementary file 1 — Supplementary Info [file 41598_2018_37377_MOESM1_ESM.pdf]

SUPPLEMENTARY INFORMATION

Modernization of Golgi staining techniques for high-resolution, 3-dimensional imaging of individual neurons.

Katlijn Vints[1,2], Dorien Vandael[1,2], Pieter Baatsen[1,2], Benjamin Pavie[2,3], Frank Vernaillen[3,4], Nikky Corthout[2,3], Vasily Rybakin[5], Sebastian Munck[2,3] & Natalia V Gounko[1,2*]

[1]VIB-KU Leuven Center for Brain & Disease Research, Electron Microscopy Platform & VIB-Bioimaging Core, O&N4 Herestraat 49 box 602, 3000 Leuven, Belgium.
[2]KU Leuven Department of Neurosciences, Leuven Brain Institute, O&N4 Herestraat 49 box 602, 3000 Leuven, Belgium.
[3]VIB-KU Leuven Center for Brain & Disease Research, Light Microscopy Expertise Unit & VIB Bioimaging Core, O&N4 Herestraat 49 box 602, 3000 Leuven, Belgium.
[4]VIB Bioinformatics Core, Rijvisschestraat 126 3R, 9052 Gent, Belgium.
[5] Rega Institute, Department of Microbiology and Immunology KU Leuven, Herestraat 49 box 1044, 3000 Leuven, Belgium.

*Corresponding author:

Prof. Natalia Gounko
VIB – KU Leuven Center for Brain & Disease Research
KU Leuven Department of Neurosciences, Leuven Brain Institute
Herestraat 49, 3000 Leuven, Belgium.
Tel. +32 16 374564
Fax +32 16 330827
E-Mail: natalia.gunko@kuleuven.vib.be

Supplementary Figure S1. Golgi-Cox method and plaque staining wild type mice: negative control images for Fig. 5. A 200-µm coronal brain section of a 25-month-old WT female mouse (age/sex-matched control for the APP knock-in mice (APP$^{NL\text{-}G\text{-}F}$)), impregnated with the Golgi-Cox method and stained for amyloid-β plaques with thioflavin-S. Left, Golgi-Cox stain; middle, plaque stain; right, increased gain for thioflavin-S. No plaques have been observed (middle) even with strongly increased gain in the thioflavin-S channel. Scale bars: 150 µm.

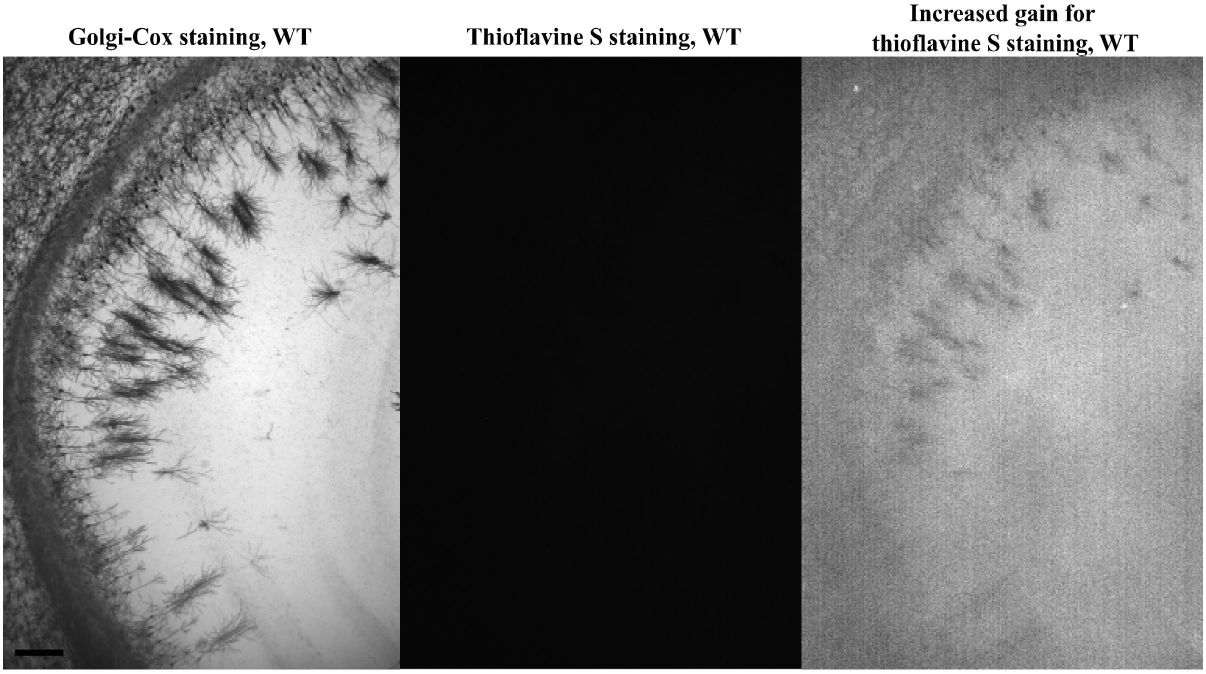

SUPPLEMENTARY VIDEO LEGENDS

All movies can be accessed by following the link below http://bioimagingcore.be/videos/

Supplementary Video 1. Open "sandwich" of original Golgi after silver impregnation and brush away the precipitation from vibratome sections.

Supplementary Video 2. OPT imaging of cleared hippocampus impregnated with the original Golgi method.

Supplementary Video 3. Step-by-step tutorial on using our custom Golgi-Stain plugin for ImageJ. At 5”-53”, semi-automatic segmentation of an impregnated hippocampal neuron in EM images, using the plugin described in this paper, with the explanation of user-defined parameters. At 53”-1’53”, segmentation followed by an example of simple post-processing and basic 3D rendering in ImageJ.

Supplementary Video 4. 3D reconstruction of semi-automatically segmented neuron. At 2”-6”, a stack of images cut with serial BF-SEM. The soma of a Golgi-stained neuron (dark) is clearly visible in the left top corner at 5”-6”. At 6”-26”, Golgi-stained cells, semi-automatically segmented using the ImageJ plugin, reconstructed and visualized in Amira. In orange, reconstructed 3D model of all black neurons, with one cell highlighted as a neuron of interest. At 23”-25”, a gap, marked with asterisk, in the Golgi visualization, indicates the ROI that has been cut manually. At 28”-49”, the third dendrite of a hippocampal granular cell and a presynaptic bouton imaged using TEM, followed by manual alignment, segmentation, reconstruction and visualization in Amira. Orange, dendrite of a Golgi-impregnated cell; yellow, presynaptic terminal; green, mitochondria; blue, synaptic vesicles; pink, postsynaptic density.
