# Supplementary material for: Modernization of Golgi staining techniques for high-resolution, 3-dimensional imaging of individual neurons
Source: Sci Rep. 2019 Jan 15;9:130. doi: 10.1038/s41598-018-37377-x (PMC6333844; doi:10.1038/s41598-018-37377-x)
Supplement: Supplementary file 6 — golgi-stain-1.2.0 plugin for ImageJ [file 41598_2018_37377_MOESM6_ESM.zip › SREP-18-38797-T_Vints_et_al_Title_page.docx]

SREP-18-38797

Modernization of Golgi staining techniques for high-resolution, 3-dimensional imaging of individual neurons.

Vints K, Vandael D, Baatsen P, Pavie B, Vernaillen F, Corthout N, Rybakin R, Sebastian Munck S, Gounko NV
